# 1D ^13^C-NMR Data as Molecular Descriptors in Spectra — Structure Relationship Analysis of Oligosaccharides

**DOI:** 10.3390/molecules17043818

**Published:** 2012-03-28

**Authors:** Florbela Pereira

**Affiliations:** CQFB and REQUIMTE, Departamento de Química, Faculdade de Ciências e Tecnologia, Universidade Nova de Lisboa, 2829-516 Caparica, Portugal; Email: florbela.pereira@fct.unl.pt; Tel.: +351-21-294-8300; Fax: +351-21-294-8550

**Keywords:** machine learning techniques, Random Forest, classification tree, CPGNN, ^13^C-NMR, oligosaccharides, disaccharides, trisaccharides

## Abstract

Spectra-structure relationships were investigated for estimating the anomeric configuration, residues and type of linkages of linear and branched trisaccharides using ^13^C-NMR chemical shifts. For this study, 119 pyranosyl trisaccharides were used that are trimers of the α or β anomers of D-glucose, D-galactose, D-mannose, L-fucose or L-rhamnose residues bonded through α or β glycosidic linkages of types 1→2, 1→3, 1→4, or 1→6, as well as methoxylated and/or *N*-acetylated amino trisaccharides. Machine learning experiments were performed for: (1) classification of the anomeric configuration of the first unit, second unit and reducing end; (2) classification of the type of first and second linkages; (3) classification of the three residues: reducing end, middle and first residue; and (4) classification of the chain type. Our previously model for predicting the structure of disaccharides was incorporated in this new model with an improvement of the predictive power. The best results were achieved using Random Forests with 204 di- and trisaccharides for the training set—it could correctly classify 83%, 90%, 88%, 85%, 85%, 75%, 79%, 68% and 94% of the test set (69 compounds) for the nine tasks, respectively, on the basis of unassigned chemical shifts.

## 1. Introduction

Carbohydrates play key roles in many biological processes, however their functions and the mechanisms of these processes are still not completely known. As a result, carbohydrates remain the least exploited among the three major classes of biomolecules. Additionally, the building blocks of polysaccharides sequences is significantly larger than for the residues in proteins or nucleic acids, more than 30 monosaccharides have been identified in mammalian polysaccharides and even more so than 500 in bacterial polysaccharides [1]. The rich variety of possible linkages positions between the monosaccharides as well as their stereochemistry increase the difficulty of their structural analysis.

The elucidation of carbohydrate structures from the simplest monosaccharides to the most complex branched polysaccharides is crucial to understand the wide-ranging functions of carbohydrates in biological systems. Their biological activities are mainly due to their surface properties which depend on their structure and conformation.

Nowadays, NMR spectroscopy has become a sophisticated and powerful analytical technology that has found a variety of applications in many disciplines of scientific research, medicine, and various industries. Modern NMR has been emphasizing the application in biomolecular systems and plays an important role in structural biology. The determination of the linkage type and anomeric configuration, as well as assigning the monomer stereochemistry is frequently the main objective of an NMR study of unknown polysaccharides from biological materials. The complete analysis of carbohydrates is a complex, time-consuming process that usually makes use of a variety of 2D techniques [2,3,4], such as ^1^H-^1^H TOCSY and DQF-COSY, ^1^H-^1^H NOESY, ^1^H-^1^H ROESY, ^13^C-^1^H HSQC, and ^13^C-^1^H HMQC or HMBC. The challenge and the same time the complexity of these problems have led to the development of computerized approaches [5,6,7].

Jansson and co-workers [8,9,10,11] developed the Computer Assisted SPectrum Evaluation of Regular polysaccharides (CASPER) program [12], which provides a structural analysis of linear oligo- and polysaccharides, as well as, branched counterparts using ^1^H and ^13^C chemical shift data and ^1^*J*_CH_ or ^3^*J*_HH_ scalar coupling constants. CASPER generates the predicted ^1^H and ^13^C chemical shifts from input of the oligo- or polysaccharide chemical structure (*i.e.*, constituent monosaccharides, linkage types as well as methoxylated derivatives and anomeric configurations). In addition, the predicted chemical shifts are ranked according to the lowest average total difference between predicted and experimental chemical shifts. The ^1^H and ^13^C chemical shift data can be also used as input and then the CASPER output displays all possible chemical structures match this data. These structures are evaluated by comparison of the structures based on the input NMR data and the predicted chemical shifts. However, the structural determination of an oligo- or polysaccharide using CASPER requires, besides to the NMR data, information about the residues and their linkages.

Many research works cite the use of NMR to determine 3-D structure of macromolecules and in the detection, identification and quantification of potential drug compounds. However, there are quite few studies reporting the use of NMR data in quantitative structure–activity relationships (QSAR) modeling [13,14] or/and spectra-structure correlations [7,15,16,17,18,19]. There is a huge source of information that has been created in the last years from the NMR data. All this information is processed almost only by human learning process. We think that is possible to use this kind of information as an input in machine learning techniques with a high improvement of the results, since it is possible that the human discrimination could miss out important and subjacent information. The NMR data could be seen as a fingerprint of the 3D chemical structure and as well as fingerprint of the electronic and surface properties of a molecule. In accordance with our previous work [7] in which, spectra-structure correlation models to predict the anomeric configuration, type of linkage and residues for disaccharides from unassigned list of ^13^C chemical shifts were built with high accuracy.

The aim of this study is the development of new computational tools for structural elucidation of oligosaccharides, such as di- and trisaccharides, using 1D ^13^C-NMR data. This approach is complementary to the program CASPER because it allows the prediction of the anomeric configuration, residues and type of linkages in oligosaccharides using only the ^13^C-NMR chemical shifts sorted in ascending order (unassigned chemical shifts). In fact, the structure determination of an oligosaccharide using CASPER requires, besides to the ^13^C-NMR chemical shifts, information about the residues and their linkages.

## 2. Results and Discussion

Different representative machine learning techniques, such as RF, CT and CPGNN, were compared to build a quantitative spectra–structure relationships model to predict: (1) the three anomeric configurations; (2) the two type of linkages; (3) the three residues; and (4) the chain type of trisaccharides from 1D ^13^C-NMR descriptors. The results for internal cross-validation (10-fold cross-validation with CPGNN and out-of-bag estimation with RF on training set) and external validation (on test set) are presented in Table 1 and Table 2.

**Table 1:** RF, CT and CPGNN predictions of the anomeric configurations, type of linkages, residues and chain type in trisaccharides from 1D ^13^C-NMR descriptors.

			RF ^a^			CT			CPGNN ^b^		
			Training set / Test set								
Model	Classes	Size	Correct pred.	Sensitivity ^c^	Specificity ^d^	Correct pred.	Sensitivity	Specificity	Correct pred.	Sensitivity	Specificity
**Ano_F^1^**	**A (** **α)**	46/12	33/6	0.71/0.50	0.75/0.67	43/7	0.93/0.58	0.78/0.58	27/5	0.59/0.42	0.59/0.56
	**B (** **β)**	46/15	35/12	0.76/0.8	0.73/0.67	34/10	0.74/0.67	0.92/0.67	27/11	0.59/0.73	0.59/0.61
**Ano_S^2^**	**A (** **α)**	46/12	45/12	0.98/1	0.98/0.92	38/5	0.83/0.42	0.80/0.67	30/5	0.65/0.42	0.62/0.56
	**B (** **β)**	46/15	45/14	0.98/0.93	0.98/1	37/10	0.81/0.50	0.82/0.59	28/11	0.61/0.73	0.64/0.61
**Ano_R^3^**	**A (** **α)**	46/16	34/12	0.74/0.75	0.77/0.92	38/11	0.83/0.69	0.93/1	23/8	0.5/ 0.5	0.74/0.89
	**B (** **β)**	46/11	36/10	0.78/0.91	0.75/0.71	43/11	0.93/1	0.84/0.69	38/10	0.83/0.91	0.62/0.56
**F_Link^4^**	**A (1→2)**	33/13	30/10	0.91/0.77	0.83/1	28/10	0.85/0.77	0.78/0.62	8/4	0.24/0.31	0.53/0.8
	**B (1→3)**	27/7	22/7	0.81/1	0.85/0.78	23/4	0.85/0.57	0.72/0.57	18/6	0.67/0.86	0.34/0.35
	**C (1→4)**	16/4	11/2	0.69/ 0.5	0.92/1	8/0	0.5/0	0.67/0	6/3	0.38/0.75	0.28/0.6
	**D (1→6)**	16/3	15/3	0.94/1	0.83/0.5	12/3	0.75/1	1/1	0/0	0/0	0/0
**S_Link^5^**	**A (1→2)**	8/1	1/0	0.12/0	1/0	0/0	0/0	0/0	0/0	0/0	0/0
	**B (1→3)**	17/12	9/7	0.53/0.58	1/1	12/8	0.70/0.67	0.86/1	7/6	0.41/0.5	0.32/0.86
	**C (1→4)**	51/13	50/13	0.98/1	0.74/0.72	47/11	0.92/0.85	0.75/0.73	39/12	0.76/0.92	0.57/0.6
	**D (1→6)**	16/1	13/1	0.81/1	0.93/0.5	13/1	0.81/1	0.87/0.25	0/0	0/0	0/0
**Red_end^6^**	**A (Glc)**	26/17	21/14	0.81/0.82	0.6/1	18/10	0.69/0.59	0.75/0.77	6/3	0.23/0.18	1/1
	**B (Gal)**	18/5	8/5	0.44/1	0.5/0.71	8/2	0.44/0.4	0.89/1	6/2	0.33/0.4	0.17/0.14
	**C (Man)**	18/3	9/2	0.5/0.67	0.64/0.67	11/1	0.61/0.33	0.61/0.2	9/1	0.5/0.33	0.24/0.12
	**D (Rha)**	17/2	12/2	0.70/1	0.92/0.67	16/1	0.94/0.5	0.84/0.25	3/2	0.23/1	0.25/1
	**E (Fuc)**	13/0	10/0	0.77/---	0.71/---	13/0	1/---	0.59/---	1/0	0.08/---	1/---
**M_residue^7^**	**A (Glc)**	26/6	19/5	0.73/0.83	0.61/0.56	20/3	0.77/0.5	0.83/0.33	7/3	0.27/0.5	0.78/0.6
	**B (Gal)**	19/5	9/4	0.47/0.8	0.64/0.8	8/1	0.42/0.2	0.53/0.17	11/2	0.58/0.4	0.34/0.2
	**C (Man)**	19/5	9/4	0.47/0.8	0.43/0.8	12/2	0.63/0.4	0.63/0.67	8/0	0.42/0	0.35/0
	**D (Rha)**	12/4	6/3	0.5/0.75	0.6/1	10/3	0.83/0.75	0.53/0.75	7/3	0.58/0.75	0.25/0.5
	**E (Fuc)**	16/7	9/4	0.56/0.57	0.56/0.8	11/4	0.69/0.57	0.73/0.8	0/1	0/0.14	0/1
**F_residue^8^**	**A (Glc)**	22/9	18/6	0.82/0.67	0.86/0.67	15/5	0.68/0.56	0.83/0.62	10/2	0.45/0.22	0.91/1
	**B (Gal)**	18/3	14/2	0.78/0.67	0.82/0.28	11/1	0.61/0.33	0.69/0.17	8/2	0.44/0.67	0.38/0.17
	**C (Man)**	16/6	9/2	0.56/0.33	0.56/1	13/5	0.81/0.83	0.59/0.83	6/2	0.38/0.33	0.21/0.5
	**D (Rha)**	19/4	14/3	0.74/0.75	0.7/0.75	12/3	0.63/0.75	0.63/1	8/2	0.42/0.5	0.28/0.28
	**E (Fuc)**	17/5	15/3	0.88/0.6	0.83/0.6	14/3	0.82/0.6	0.82/0.75	1/1	0.06/0.2	0.33/0.5
**Chain_Type**	**A (LT)**	39/8	29/7	0.74/0.88	0.83/0.88	37/8	0.95/1	0.80/0.67	26/6	0.67/0.75	0.81/1
	**B (BT)**	53/19	47/18	0.89/0.95	0.82/0.95	44/15	0.83/0.79	0.96/1	47/19	0.89/1	0.78/0.90

^a^ Out-of-bag; ^b^ 10-Fold cross-validation; ^c^ Ratio of true positives to the sum of true positives and false negatives; ^d^ Ratio of true positives to the sum of true positives and false positives; ^1^ Anomeric configuration of the first unit; ^2^ Anomeric configuration of the second unit; ^3^ Anomeric configuration of the reducing end; ^4^ First linkage type; ^5^ Second linkage type; ^6^ Reducing end; ^7^ Middle residue; ^8^ First residue.

**Table 2:** Mean predictability of RF, CT and CPGNN predictions of the anomeric configurations, type of linkages, residues and chain type in trisaccharides from 1D ^13^C-NMR.

		Mean Predictability (%) ^a^					
		Training set ^b^			Test set		
	Model	RF	CT	CPGNN	RF	CT	CPGNN
Anomeric Configurations	**Ano_F^1^**	73.91	83.70	58.70	65.00	62.5	57.50
	**Ano_S^2^**	97.83	81.52	63.04	96.67	54.17	57.50
	**Ano_R^3^**	76.09	88.04	66.30	82.95	84.38	70.45
Linkage Types	**F_Link^4^**	83.72	73.76	32.10	81.73	58.52	47.87
	**S_Link^5^**	61.18	61.00	29.41	64.58	62.82	35.58
Residues	**Red_end^6^**	64.54	73.78	26.35	87.25	45.54	47.74
	**M_residue^7^**	54.81	66.85	37.05	75.10	48.43	41.25
	**F_residue^8^**	75.55	71.21	35.08	60.33	61.44	43.06
	**Chain_Type**	81.52	88.94	77.67	91.12	89.47	87.50

^a^ Average sensitivity of the trisaccharide classes; ^b^ 10-Fold cross-validation with CPGNN and out-of-bag estimation with RF on training set; ^1^ Anomeric configuration of the first unit; ^2^ Anomeric configuration of the second unit; ^3^ Anomeric configuration of the reducing end; ^4^ First linkage type; ^5^ Second linkage type; ^6^ Reducing end; ^7^ Middle residue; ^8^ First residue.

**Figure 1:**
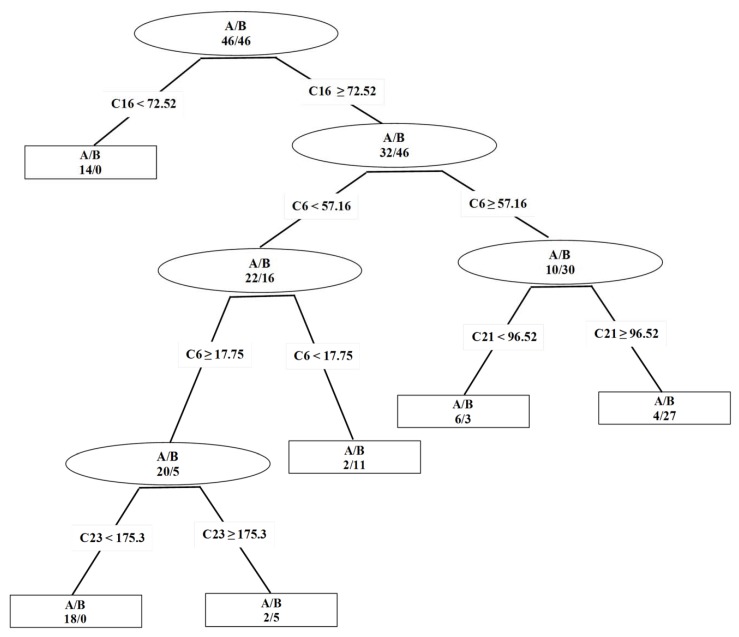
Representation of the classification tree derived with CART algorithm to distinguish the reducing end anomeric configuration of 92 trisaccharides (training set).

The random forest method showed an improved prediction performance compared to a single classification tree and CPGNN method to predict the anomeric configurations, type of linkages, residues, and chain type of trisaccharides for test set (Table 2). However, a single tree was able to predict the reducing end anomeric configuration of the training set and external data set with a mean predictability of 88.0% and 84.4% (for the α anomer: sensitivity = 0.69, specificity = 1 and for the β anomer: sensitivity = 1, specificity = 0.69) respectively, using unassigned chemical shifts (Table 1 and Table 2). The performance of the reducing end anomeric tree was even slightly superior to RF in terms of correct predictions for the training set, and similar for the predictions of the test set. A graphical representation of the reducing end anomeric tree was presented in Figure 1. Four descriptors were chosen by the tree, although one descriptor was used twice, C6 (6th chemical shift). All of these descriptors are identified as the ten most important spectral descriptors by the RF revealed by the Gini parameter—C16 (16th chemical shift), C21 (21st chemical shift) and C23 (23rd chemical shift).

In addition, it was possible to infer important rules derived by the reducing end anomeric classification tree. Rules such as: (1) at the first node, for 16th chemical shift <72.52 ppm, all the trisaccharides are α anomers and almost all without D-glucose monomer in any position (only one trisaccharide has a D-glucose unit), otherwise (16th chemical shift ≥72.52 ppm.) the reducing end, second unit or first unit of all trisaccharides may be D-glucose monomer or not; (2) at the second node, for 6th chemical shift ≥57.16 ppm, most of trisaccharides have only as reducing, middle and first residue a D-glucose, a D-galactose or a D-mannose monomer (eight trisaccharides have a L-fucose or L-rhamnose monomer units), otherwise (6th chemical shift <57.16 ppm) all trisaccharides have at least a L-fucose or a L-rhamnose monomer; (3) at the third node, for 21st chemical shift ≥96.52 ppm, nearly all the trisaccharides are β anomers without a L-fucose or a L-rhamnose monomer, otherwise (21st chemical shift <96.52 ppm) almost all the trisaccharides are non-methoxylated linear α anomers; (4) at the fourth node, for the 23rd chemical shift ≥175.3 ppm, almost all trisaccharideas are linear β anomers, otherwise (23rd chemical shift <175.3 ppm) all trisaccharides are methoxylated branched α anomers with at least one L-fucose or L-rhamnose residue.

In order to compare the nine models of classification built with RF and CT, just an experiment was performed with a counterpropagation neural network (CPGNN) on the basis of unassigned ^13^C chemical shifts. In spite of this advantage of the CPGNN as compared to CT and RF methods, its predictive power is lower than those for all classes with no exceptions within internal validation (*i.e.*, 10-fold cross-validation procedure with training set) and external validation (*i.e.*, with test set, Table 1 and Table 2).

After the exploration of models derived with trisaccharides, we investigated the inclusion in this new model of our previously model for predicting the structure of 154 pyranosyl disaccharides using unassigned ^13^C-NMR chemical shifts (Table 3). RF was used due to its best performance in previous experiments.

**Table 3:** RF predictions of the anomeric configurations, type of linkages, residues and chain type in 204 di- and trisaccharides for the training set and 69 for the test set using 1D ^13^C-NMR descriptors.

		Training set / Test set				
Model	Classes	Size	Correct pred.	Sensitivity ^a^	Specificity ^b^	Mean Predictability ^c^ (%)
**Ano_F^1^**	**A (** **α)**	105/30	89/25	0.85/0.83	0.88/0.78	86.32/82.69
	**B (** **β)**	99/39	87/32	0.88/0.82	0.84/0.86	
**Ano_S^2^**	**A (** **α)**	46/12	38/10	0.83/0.83	0.74/0.83	84.78/90
	**B (** **β)**	46/15	33/13	0.72/0.87	0.80/0.87	
	**X (NA)**	112/42	112/42	1/1	1/1	
**Ano_R^3^**	**A (** **α)**	102/39	84/31	0.82/0.79	0.88/0.94	85.78/88.08
	**B (** **β)**	102/30	91/28	0.89/0.93	0.83/0.78	
**F_Link^4^**	**A (1→2)**	33/13	30/10	0.91/0.77	0.79/1	84.99/85.38
	**B (1→3)**	27/7	21/7	0.78/1	0.88/0.78	
	**C (1→4)**	16/4	11/2	0.69/0.5	0.85/0.67	
	**D (1→6)**	16/3	14/3	0.88/1	0.82/0.6	
	**X (NA)**	112/42	112/42	1/1	1/1	
**S_Link^5^**	**A (1→2)**	36/13	22/10	0.61/0.77	0.88/0.83	82.32/85.16
	**B (1→3)**	48/21	38/15	0.79/0.71	0.84/0.94	
	**C (1→4)**	71/26	69/24	0.97/0.92	0.748/0.77	
	**D (1→6)**	49/9	45/9	0.92/1	0.98/0.9	
**Red_end^6^**	**A (Glc)**	72/38	60/33	0.83/0.87	0.71/0.75	75.70/74.96
	**B (Gal)**	58/13	39/9	0.67/0.69	0.72/0.75	
	**C (Man)**	44/16	37/7	0.73/0.44	0.86/0.7	
	**D (Rha)**	17/2	12/2	0.70/1	0.92/0.67	
	**E (Fuc)**	13/0	11/0	0.87/---	0.69/---	
**M_residue^7^**	**A (Glc)**	26/6	20/5	0.77/0.83	0.69/0.56	62.27/79.25
	**B (Gal)**	19/5	9/4	0.47/0.8	0.5/0.8	
	**C (Man)**	19/5	7/4	0.37/0.8	0.37/0.8	
	**D (Rha)**	12/4	6/3	0.5/0.75	0.67/1	
	**E (Fuc)**	16/7	10/4	0.62/0.57	0.59/0.8	
	**X (NA)**	112/42	112/42	1/1	1/1	
**F_residue^8^**	**A (Glc)**	74/33	65/31	0.88/0.94	0.86/0.76	79.64/67.50
	**B (Gal)**	44/7	31/2	0.70/0.28	0.74/0.33	
	**C (Man)**	50/20	39/12	0.78/0.6	0.78/1	
	**D (Rha)**	19/4	14/3	0.74/0.75	0.67/0.75	
	**E (Fuc)**	17/5	15/4	0.88/0.8	0.88/0.67	
**Chain_Type**	**A (LT)**	39/8	28/7	0.72/0.88	0.82/0.88	86.82/94.08
	**B (BT)**	53/19	47/18	0.89/0.95	0.81/0.95	
	**X (NA)**	112/42	112/42	1/1	1/1	

^a^ Ratio of true positives to the sum of true positives and false negatives; ^b^ Ratio of true positives to the sum of true positives and false positives; ^c^ Average sensitivity of the trisaccharides classes; ^1^ Anomeric configuration of the first unit; ^2 ^Anomeric configuration of the second unit; ^3^ Anomeric configuration of the reducing end; ^4^ First linkage type; ^5^ Second linkage type; ^6^ Reducing end; ^7^ Middle residue; ^8^ First residue.

When the trisaccharides and disaccharides models were taken together the performance of seven and eight of the nine tasks [*i.e.*, (1) anomeric configuration of the first unit, second unit and reducing end; (2) type of first and second linkages; (3) reducing end, middle and first residue; and (4) chain type of di- and trisaccharides] were improved for the test set and training set, respectively, see Table 1–Table 3. In fact the di- and trisaccharides model showed an improved prediction performance compared to the trisaccharides model in terms of correct predictions for test set—It could classify without any error or with only one error for the nine tasks 52% of the trisaccharides (14 of 27 trisaccharides) as compared with 37% for the trisaccharides model (10 of 27 trisaccharides). As expected with the increase of the system complexity, for the prediction of disaccharides in the new model was obtained a lower result in terms of correct predictions for the test set to that obtained with the disaccharides model (*i.e.*, our previous model)—It could classify without any error or with only one error for the nine tasks 83% of the disaccharides (35 of 42 disaccharides) as compared with 95% for the disaccharides model (40 of 42 disaccharides). Although the decrease of the predictive power of the new model for the disaccharides, it still showing for all tasks a mean predictability great than or equal to 67% for the test set (93%, 100%, 90%, 100%, 88%, 67%, 100%, 81% and 100% for the anomeric configuration of the first unit, anomeric configuration of the second unit, anomeric configuration of the reducing end, first linkage type, second linkage type, reducing end, middle residue, first residue and chain type of 42 disaccharides, respectively).

The random forest algorithm for classification can give two measures of importance for the descriptors used in growing trees, the Mean Decrease in Accuracy and Mean Decrease in Gini. The ten most important descriptors using RF for trisaccharides model and oligosaccharides model (*i.e.*, di- and trisaccharides model), as well as the descriptors selected by the CT in the trisaccharides model were analyzed—Table 4. From the analysis it was evident that the 23rd, 22nd, 21st and 20th chemical shift descriptors have gained importance on di- and trisaccharide approach as compared with trisaccharides approach. These descriptors correspond mostly to the chemical shift of the anomeric carbon atoms. Although for the *N*-acetylated amino sugar derivatives the 23rd descriptor corresponds to the chemical shift of the carbonyl carbon atom in 2-acetamide group.

**Table 4:** Comparison of the ten most important descriptors by RF in the trisaccharides and oligosaccharide models and the selected descriptors by CT in the trisaccharides model.

Model		RF		CT
		Trisaccharides	Di- and trisaccharides	
**Ano_F^1^**		C12; C11; C9; C22; C14; C13; C23; C15; C16; C17	C23; C19; C20; C12; C15; C18; C21; C13; C11; C22	C12; C9 (2×); C21
**Ano_S^2^**		C15; C14; C13; C22; C16; C6; C10; C23; C17; C7	C12; C6; C10; C9; C8; C11; C7; C21; C13; C14	C14; C6; C22
**Ano_R^3^**		C16; C6; C21; C10; C14; C18; C11; C17; C5; C8	C22; C18; C16; C20; C19; C17; C14; C23; C21; C6	C16; C6 (2×); C21; C2
**F_Link^4^**	C8; C7; C20; C6; C19; C23; C10; C14; C16; C11		C8; C7; C6; C10; C21; C12; C11; C9; C20; C13	C8; C20; C7; C11; C6; C17
**S_Link^5^**	C8; C7; C19; C6; C22; C20; C5; C18; C9; C23		C21; C13; C14; C15; C8; C22; C20; C12; C23; C19	C8 (2×); C22; C17
**Red_end^6^**	C6; C5; C22; C19; C20; C7; C9; C10; C14; C18		C22; C15; C14; C16; C20; C13; C19; C21; C18; C17	C6; C12; C5; C20; C23
**M_residue^7^**	C6; C7; C15; C5; C16; C11; C23; C9; C10; C18		C10; C7; C6; C9; C8; C12; C11; C21; C15; C5	C16; C6 (2×); C7; C18; C23
**F_residue^8^**	C7; C5; C6; C10; C15; C16; C8; C9; C11; C21		C14; C12; C16; C15; C23; C17; C20; C5; C21; C13	C16; C9 (2×); C7; C8; C5
**Chain_Type**	C7; C23; C20; C14; C5; C8; C6; C21; C18; C12		C8; C21; C7; C6; C9; C10; C11; C12; C14; C23	C7; C5; C23 (2×); C8

^1^ Anomeric configuration of the first unit; ^2^ Anomeric configuration of the second unit; ^3^ Anomeric configuration of the reducing end; ^4^ First linkage type; ^5^ Second linkage type; ^6^ Reducing end; ^7^ Middle residue; ^8^ First residue.

## 3. Experimental

### 3.1. Data Set and Descriptors

A data set of 119 pyranosyl trisaccharides and their corresponding twenty three ^13^C-NMR chemical shifts were used for establishing spectra–structure relationships. Trisaccharides used are trimers of the α or β anomers of D-glucose, D-galactose, D-mannose, L-fucose or L-rhamnose residues bonded through α or β glycosidic linkages of types 1→2, 1→3, 1→4, or 1→6, as well as methoxylated and/or N-acetylated amino trisaccharides. The ^13^C-NMR chemical shifts of the test set (27 trisaccharides) were experimental values obtained from the literature [19,20,21,22,23,24,25,26] and chemical shifts of the training set were also experimental values obtained from the literature (57 trisaccharides) [20,21,22,23,24,25,26,27,28,29] as well as chemical shifts calculated by the CASPER program [12,30] (35 trisaccharides).

The training and test sets are listed in Table S1 and S2 of the Supporting Information. The chemical shifts (independent variables) were encoded as a sequence of chemical shifts sorted in ascending order. The input, 1D ^13^C descriptors, corresponds to the ^13^C chemical shifts of only one of the epimeric disaccharides (α or β configuration of the reducing end). Therefore, the model that was constructed did not consider the mutarotation process, because ^13^C chemical shifts corresponding to a single anomer (i.e., a single diastereoisomer) had been used for each one of the input objects. These chemical shifts should, however, be interpreted only qualitatively, since oligosaccharides are flexible molecules, and the measured (for the test and training sets) and calculated (for the training set) chemical shifts represent an average for all existing conformations.

The three anomeric configurations of the 119 trisaccharides result in three outputs and for each one two classes , A(α) and B (β) corresponding to the stereochemistry of the glycosidic linkage between the first and second units, glycosidic linkage between the second and third units, and the reducing end of the trisaccharide, respectively. The linkage discrimination of these trisaccharides was also achieved using two outputs and for each one four classes, A (1→2), B (1→3), C (1→4) and D (1→6), corresponding to the first and second linkage positions of the trisaccharide. The three monomers generate three outputs and for each one five classes, A (Glc), B (Gal), C (Man), D (Rha), and E (Fuc), corresponding to the reducing end, middle and first residue of the trisaccharide. The chain type of these trisaccharides was also achieved using two classes, A (linear trisaccharide—LT), B (branched trisaccharide—BT).

Our previously model for predicting the structure of 154 pyranosyl disaccharides using unassigned ^13^C-NMR chemical shifts [7] was incorporated in this new model in order to evaluate its predictive power, as well as to compare it with the results obtained using the disaccharides model. The partition between training set and test set was maintained. Therefore, this new model (*i.e.*, di- and trisaccharides model) is built with 204 di- and trisaccharides for the training set and 69 for the test set. The disacchaides training and test sets are listed in Table S3 and S4 of the Supporting Information.

The main objective of this procedure was to verify if unassigned ^13^C-NMR chemical shifts values could enable machine learning techniques, to clearly discriminate between various anomeric configurations, type of linkages, and residues of trisaccharides as well as disaccharides. As a result, building a tool that allows the users to predict the structure of oligosaccharides from the unassigned ^13^C-NMR chemical shifts could be very useful.

The validation of the CASPER program by Loß *et al.* [31] showed in many cases discrepancies between the calculated and experimental 13C chemical shifts as low as 0.2 ppm for 155 glycan compounds. These values have the same range of differences between measurements from different laboratories resulting from slightly dissimilar experimental conditions. Moreover, in our previous work with disaccharides [7], the calculated ^13^C-NMR chemical shifts of the training set using the CASPER program were validated with experimental values obtained from the literature for the test set and a maximum error and mean RMS (root mean square) error of 0.95 ppm and 0.15 ppm, respectively, was obtained.

### 3.2. Selection of Training and Test Sets for the Trisaccharides Model

The whole data set was divided into a training set of 92 compounds and a test set of 27 compounds, which were used for the development and external validation of the quantitative spectra-structure relationships models. The approximate 3:1 partition was assisted by a Kohonen Self-Organizing Map (SOM) [32] in such way that both sets span the chemical diversity of the data set. The 119 compounds were mapped on a SOM on the basis of unassigned twenty three ^13^C-NMR chemical shifts (independent variables). No information concerning the structure was used. A trend for clustering according to structural classes of compounds such as D-glucans trisaccharides (consisting of three monomers of D-glucose) was observed. Compounds belonging to the various clusters were selected for the test set from singly occupied neurons.

### 3.3. Random Forest (RF) [33,34,35]

A RF is an ensemble of unpruned classification trees created by using bootstrap samples of the training data. The best split at each node was defined among a randomly selected subset of descriptors. It is a high-dimensional nonparametric method that usually works well on large numbers of descriptors. Prediction is made by a majority vote of the classification trees in the forest. It has been shown that the method is extremely accurate in a variety of applications [34]. Additionally, performance is internally assessed with the prediction error for the objects left out, called out-of-bag (OOB) data, in the bootstrap procedure (internal cross-validation or OOB estimation). The method quantifies the importance of a descriptor by the increase in misclassification occurring when the values of the descriptor are randomly permuted, correlated with the mean decrease in accuracy parameter, or by the decrease in a node’s impurity every time the descriptor is used for splitting, correlated with mean decrease in Gini parameter. RFs also assign a probability to every prediction on the basis of the number of votes obtained by the predicted class. A measure of similarity between two objects can be calculated from the number of trees in the ensemble that classify the two objects in the same terminal node. Therefore, it is a supervised method because such comparison relies on the descriptors that were chosen by the forest to build the model. In this study, RFs were grown with the R program, version 2.12.1, [36] using the Random Forest library [37]. RFs were trained for the classification of: (1) anomeric configurations; (2) type of linkages; and (3) residues of di- and trisaccharides on the basis of their unassigned ^13^C-NMR chemical shifts (independent variables). The number of trees in a RF was set to 1,000.

### 3.4. Classification Tree (CT) [35,38]

Nine models of classification were built, and for each model a single classification tree was investigated to predict: (1) anomeric configuration of the first unit, second unit and reducing end; (2) type of first and second linkages; (3) reducing end, middle and first residue; and (4) chain type of trisaccharide. This was grown with the CART algorithm [38], which was different from the trees in the RFs. A classification tree is sequentially constructed by partitioning objects from a parent node into two child nodes. Each node is produced by a logical rule, usually defined for a single descriptor, where objects below a certain descriptor’s value fall into one of the two child nodes, and objects above fall into the other child node. The prediction for an object reaching a given terminal node is obtained by a majority vote of the objects (in the training set) reaching the same terminal node. The entire procedure comprises three main steps. First, an entire tree is constructed by data splitting into smaller nodes; each split produced is evaluated by an impurity function, which decreases as long as the new split permits the child node’s content to be more homogeneous than the parent node, which serves to minimize the Gini index. Secondly, a set of smaller, nested trees is obtained by the obliteration (pruning) of certain nodes of the tree obtained in the first step, therefore minimizing the entropy. The selection of the weakest branches is based on a cost– complexity measure that decides which subtree, from a set of subtrees with the same number of terminal nodes, has the lowest (within node) error. In this study, a classification tree was grown with the R program, version 2.12.1, [36] using the RPART library with the default parameters.

### 3.5. Counterpropagation Neural Network (CPGNN) [39]

A CPGNN consists of a Kohonen Self-Organizing Map (Kohonen SOM) [32] linked to an output layer of neurons aligned with the Kohonen layer. A Kohonen SOM distributes objects over a 2D surface (a grid of neurons) in such a way that objects bearing similar descriptors are mapped onto the same or adjacent neurons. The input data are stored in the two dimensional grid of neurons, each containing as many elements (weights) as there are input variables (twenty three ^13^C NMR chemical shifts). The nine output data (the three anomeric configurations, the two linkage types, the three residues and the chain type) are stored in the output layer that acts as a look-up table. CPNNs of toroidal topology and size 11 × 11 (number of neurons approximately 1.3 times the number of training cases) were trained with default parameters. The training was performed over 100 cycles. CPNNs were implemented with an in-house developed Java application derived from the JATOON Java applets [40,41].

## 4. Conclusions

The results indicate that machine learning techniques can be trained to predict the three anomeric configurations, the two types of linkages, the three residues and the chain type for linear or branched trisaccharides from the unassigned list of twenty three ^13^C chemical shifts with acceptable accuracy. The random forest method showed improved prediction performance compared to a single classification tree and a counterpropagation neural network to predict the nine tasks of 119 pyranosyl trisaccharides. Our previously model for predicting the structure of disaccharides was incorporated in this new model with an improvement of the predictive power.

Without the input of trisaccharide stereochemical data it was possible through the 1D ^13^C unassigned chemical shifts predict the three anomeric conformations corresponding to the stereochemistry of two glycosidic linkages and the reducing end of trisaccharide, as well as small changes in the stereochemistry of each one of residues on the trimer. For one side, the monomers D-mannose and D-galactose are epimers of D-glucose, they differ only in the stereochemistry of C-2 and C-4, respectively. And for other side, the monomers L-rhamanose and L-fucose are 6-deoxy sugars of L-mannose and L-galactose, respectively. Thus, we conclude that the results demonstrate that the 1D ^13^C chemical shifts can encode important 3D features. Therefore, the nine models built can be an important tool to predict the structure of oligosaccharides from the ^13^C-NMR chemical shifts without assigned.

Better models to predict the structure of more complex oligosaccharides as well as other natural compounds would probably require more data for calibration and can be an interesting approach in subsequent work. Applications of unassigned ^13^C-NMR chemical shifts as well as evaluations of their predictive ability as 3D molecular descriptors in quantitative structure-activity relationship (QSAR) or quantitative structure-property relationship (QSPR) analysis will be an interesting approach in subsequent work. Moreover, additional work has to be done to further investigate the application of unassigned ^13^C NMR chemical shifts as 3D molecular descriptors in quantitative structure-activity relationship (QSAR) or quantitative structure-property relationship (QSPR) analysis.

## Supplementary Materials

Supplementary materials can be accessed at: http://www.mdpi.com/1420-3049/17/4/3818/s1.
